# The effect of the fused-ring substituent on anthracene chalcones: crystal structural and DFT studies of 1-(anthracen-9-yl)-3-(naphthalen-2-yl)prop-2-en-1-one and 1-(anthracen-9-yl)-3-(pyren-1-yl)prop-2-en-1-one

**DOI:** 10.1107/S2056989018005467

**Published:** 2018-04-12

**Authors:** Dian Alwani Zainuri, Ibrahim Abdul Razak, Suhana Arshad

**Affiliations:** a X-ray Crystallography Unit, School of Physics, Universiti Sains Malaysia, 11800 USM, Penang, Malaysia

**Keywords:** chalcone, crystal structure, DFT, UV-Vis, Hirshfeld surface

## Abstract

The title chalcone compounds, C_27_H_18_O (I) and C_33_H_20_O (II), were synthesized using a Claisen–Schmidt condensation. Both compounds display an *s-trans* configuration of the enone moiety. The crystal structures feature inter­molecular C—H⋯O and C—H⋯π inter­actions. Quantum chemical analysis of density functional theory (DFT) with a B3LYP/6–311++G(d,p) basis set has been employed to study the structural properties of the compound. The effect of the inter­molecular inter­actions in the solid state are responsible for the differences between the experimental and theoretical optimized geometrical parameters. The small HOMO–LUMO energy gap in (I) (exp : 3.18 eV and DFT: 3.15 eV) and (II) (exp : 2.76 eV and DFT: 2.95 eV) indicates the suitability of these compounds for optoelectronic applications. The inter­molecular contacts and weak contributions to the supra­molecular stabilization are analysed using Hirshfeld surface analysis.

## Abstract 2

Two new anthracene chalcones, namely 1-(anthracen-9-yl)-3-(naphthalen-2-yl)prop-2-en-1-one and 1-(anthracen-9-yl)-3-(pyren-1-yl)prop-2-en-1-one, have been successfully synthesized and the effect of the different fused ring substituent system attached to the anthracene chalcone derivative investigated. These compounds show a very narrow band gap due to the large *p*-conjugated systems, making them promising candidates as optoelectronic materials. Hirshfeld surface analysis has been carried out to show the contribution of inter­molecular contacts and weak inter­actions to supra­molecular stabilization.

## Chemical context   

Naphthalene, anthracene and pyrene are three types of polycyclic aromatic hydro­carbons that consist of two, three and four fused benzene rings sharing a common side. Polyaromatic hydro­carbons or π-conjugated materials are an important class of organic compounds because of their significant conductivity properties that have led to tremendous advancements in the field of organic electronics (Li *et al.*, 2016 ▸). Most conjugated materials used in such applications rely on linear electron-rich fragments (Lin *et al.*, 2017 ▸). Furthermore, π-conjugated systems have been studied extensively for their optoelectronic properties because they give the possibility of low-cost, large-area, and flexible electronic devices. Over the past decade, significant research into new π-conjugated systems has been ongoing due to the rapidly growing number of applications in electronic devices such as semiconducting materials, organic light-emitting diodes (OLEDs; Kulkarni *et al.*, 2004 ▸) and organic field-effect trans­istors (OFETs; Torrent & Rovira, 2008 ▸; Wu *et al.*, 2010 ▸). Recently, we found that the presence of fused-ring systems at both terminal rings of chalcone derivatives to be useful in obtaining good quality single crystals with an easy-to-synthesize method. In this work, we report the synthesis and combined experimental and theoretical studies of anthracene chalcones containing a naphthalene (I) or pyrene (II) fused-ring system. Additionally, the UV–Vis absorption and Hirshfeld surface analyses are discussed.
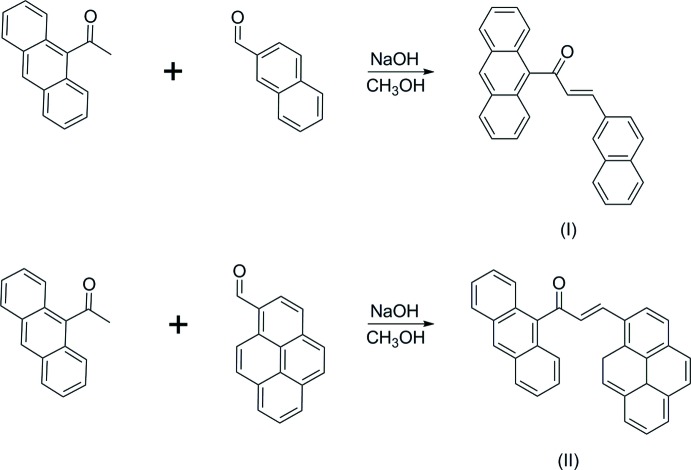


## Structural commentary   

**Figure 1:**
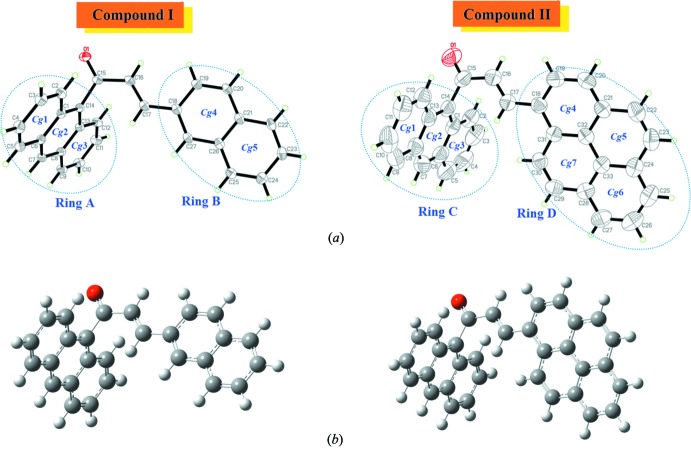
(*a*) The mol­ecular structure of compound (I) and (II), with displacement ellipsoids drawn at the 50% probability level; (*b*) the optimized structure of compound (I) and (II) at DFT/B3LYP 6–311++G(d,p).

The mol­ecular and optimized structure of compounds (I) and (II) is shown in Fig. 1 ▸. The optimization of the mol­ecular geometries leading to energy minima was achieved using DFT [with Becke’s non-local three parameter exchange and the Lee–Yang–Parr correlation functional (B3LYP)] with the 6-311++G (d,p) basis set as implemented in *Gaussian09* program package (Frisch *et al.*, 2009 ▸). From the results it can be concluded that this basis set is well suited in its approach to the experimental data. The slight deviations from the experimental values are due to the fact that the optimization is performed in an isolated condition, whereas the crystal environment affects the X-ray structural results (Zainuri *et al.*, 2017 ▸).

Compound (I) comprises a chalcone with an anthracene ring system (ring *A*) and a naphthalene ring system (ring *B*), compound (II) comprises a chalcone with an anthracene ring system (ring *C*) and a pyrene ring system (ring *D*). The enone moiety in (I) [O1/C15–C17, maximum deviation of 0.0143 (10) Å for O1] makes dihedral angles of 79.06 (11) and 8.62 (11)° with the mean planes through ring *A* [C1–C14, maximum deviation of 0.0555 (11) Å for C14] and ring *B* [C18–C27, maximum deviation of 0.037 (11) Å at C19] respectively. In compound (II), the enone moiety [O1/C15–C17, maximum deviation of 0.0364 (18) Å for O1] forms dihedral angles of 88.8 (2) and 18.3 (2)° with ring *C* [C1–C14, maximum deviation of 0.037 (3) Å for C10] and ring *D* [C18–C31, maximum deviation of 0.0236 (18) Å for C18], respectively. The large differences in the values of the dihedral angles indicate that the possibility for electronic inter­actions between the anthracene unit and the enone moiety is hindered (Jung *et al.*, 2008 ▸).

In both compounds, the C2—C3, C4—C5, C9—C10 and C11—C12 bond distances [mean value 1.3614 (18) Å for (I) and 1.351 (3) Å for (II)] are significantly shorter than the C—C bond distances in the central rings of the anthracene units [1.412 (8) and 1.403 (7) Å for (I) and (II) respectively]. This observation is consistent with an electronic structure for the anthracene units where a central ring displaying aromatic delocalization is flanked by two isolated diene units (Glidewell & Lloyd, 1984 ▸).

Both theoretical and experimental structures exist in an *s-trans* configuration of the enone moiety, with C15=O1 bond lengths of 1.2275 (14) Å (DFT: 1.22 Å) and 1.219 (2) Å (DFT: 1.22 Å) in (I) and (II), and C16=C17 bond lengths of 1.3416 (17) Å (DFT: 1.35 Å) and 1.328 (3) Å (DFT: 1.35 Å) in (I) and (II), respectively. Both compounds are twisted at the C14—C15 bond with C1—C14—C15—C16 torsion angles of 102.72 (12) and −87.9 (2)°, respectively. The corresponding torsion angles calculated by DFT are 95.94 and 95.29°, respectively. The bulkiness of the anthracene ring system gives rise to a highly twisted structure at both terminal rings. Furthermore, compounds (I) and (II) are slightly twisted at the C17—C18 bond with C16—C17—C18—C19 torsion angles of 7.35 (18)° (DFT: 0.69°) in (I) and 17.2 (13)° (DFT: 19.84°) in (II). The slight differences in the torsion angles in the two compounds is due to the formation of C—H⋯ O and C—H⋯ π inter­molecular inter­actions involving all the fused ring systems (*A*, *B*, *C* and *D*), which are not taken into consideration during the optimization process.

## Supra­molecular features   

**Figure 2:**
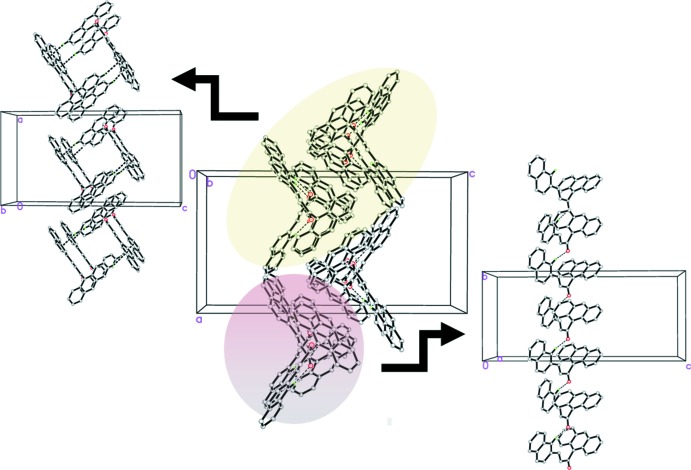
The crystal packing of compound (I) showing C—H⋯O and C—H⋯π inter­actions (dashed lines).

**Figure 3:**
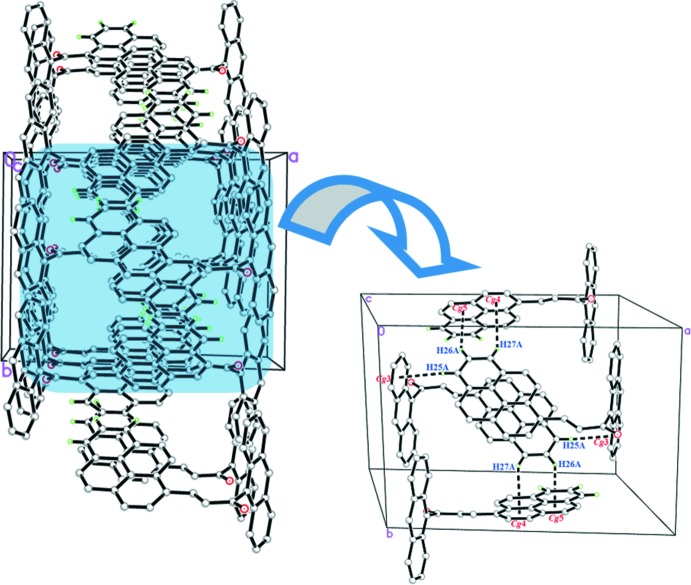
The crystal packing of compound (II) showing C—H⋯π inter­actions (dashed lines).

In compound (I), the mol­ecules are connected by weak inter­molecular C—H⋯O hydrogen bonds (Table 1 ▸) into chains propagating along the *b-*axis direction. Weak C—H⋯π inter­actions (Table 1 ▸) connect the chains into columns along the *b* axis (Fig. 2 ▸). In compound (II), mol­ecules inter­act through three kinds of C—H⋯ π inter­actions (C25—H25*A*⋯*Cg*3, C26—H26*A*⋯*Cg*5 and C27—H27*A*⋯*Cg*4; Table 2 ▸) involving the anthracene and pyrene ring systems of adjacent mol­ecules, forming a three-dimensional network (Fig. 3 ▸).

## Absorption Spectrum and Frontier Mol­ecular Orbital   

**Figure 4:**
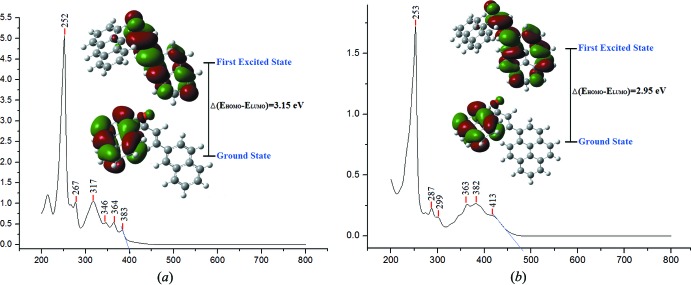
UV–Vis absorption spectra and electron distribution of the HOMO and LUMO energy levels of (*a*) compound (I) and (*b*) compound (II).

The theoretical maximum absorption wavelengths (*λ*_calc_) was obtained by time-dependent DFT (TD–DFT) calculations using B3LYP and the calculated values were compared with the experimental values. The calculations of the mol­ecular orbital geometry show that the absorption maxima of the mol­ecules correspond to the electron transition between frontier orbitals such as the transition from HOMO to LUMO. As can be seen from the UV–Vis spectra (Fig. 4 ▸), the absorption maxima values for compound (I) and (II) are found to be 383 nm, 413 nm (experimental) and 395 nm, 409 nm (theoretical), respectively. The calculated energy transitions are shifted with respect to the experiment because the calculations are confined to the gaseous equivalent whereas the observations are from the solution state. The spectroscopic data recorded show a strong cut off for compound (I) and (II) at 390 nm and 450 nm, respectively. Through an extrapolation of the linear trend observed in the optical spectra (Fig. 4 ▸), the experimental energy band gaps are 3.18 and 2.76 eV for (I) and (II) respectively. The predicted energy gaps of 3.15 and 2.95 eV are comparable to the experimental energy gaps. The energy gap for (II) is smaller because the fused ring system of the pyrene substituent has a larger π-conjugated system compared to the naphthalene fused ring system in (I). In addition, a previous study from Nietfeld *et al.* (2011 ▸) comparing the structural, electrochemical and optical properties between fused and non-fused ring compounds shows that the former have a lower band gap than other structures. The value of the optical band gaps observed for compound (I) and (II) indicate the suitability of these compounds for optoelectronic applications.

## Hirshfeld Surface Analysis   

**Figure 5:**
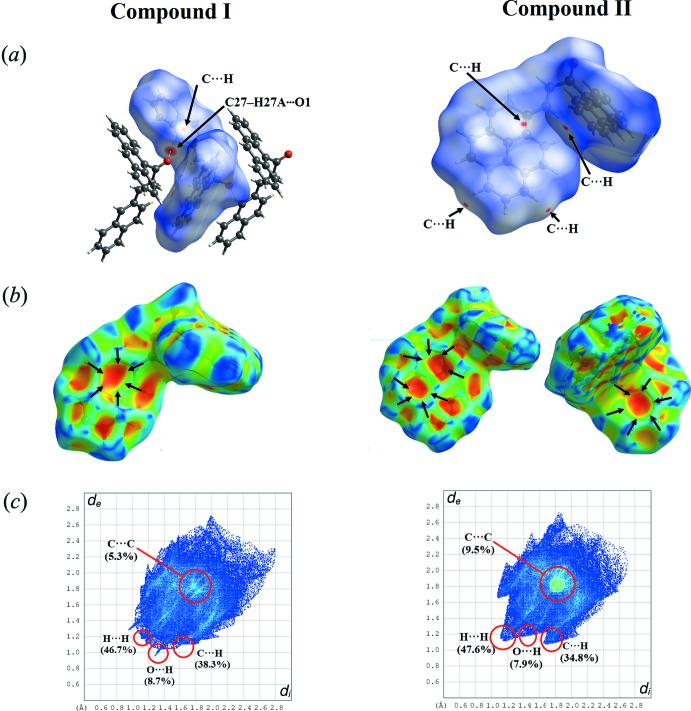
Hirshfeld surfaces for compounds (I) and (II), showing (*a*) *d*_norm_ with the red spot indicating the involvement of the C—H⋯O inter­actions; (*b*) shape index with the pale-orange spots within the black arrow indicating the C—H⋯π inter­actions; (*c*) fingerprint plots of inter­actions listing the relative percentage contribution of H⋯H, H⋯O, C⋯H and C⋯C inter­actions to the total Hirshfeld surface.

The *d_norm_* and shape-index (Wolff *et al.*, 2012 ▸) surfaces for compounds (I) and (II) are presented in Fig. 5 ▸ *a* and 5*b*, respectively. C—H⋯O and C—H⋯π contacts are shown on the *d_norm_* mapped surfaces as deep-red depression areas in Fig. 5 ▸ *a*. The C—H⋯O contacts are only present in compound (I). The C—H⋯π inter­actions are indicated through a combination of pale orange and bright-red spots, which are present on the Hirshfeld Surface mapped over the shape index surface and identified by black arrows (Fig. 5 ▸ *b*).

In the fingerprint plot (Fig. 5 ▸ *c*), the H⋯H, H⋯O, C⋯H and C⋯C inter­actions are indicated together with their relative percentage contribution. The H⋯H contacts have the largest overall contribution to the Hirshfeld surface and dominate in the crystal structure. The contribution of H⋯O/ O⋯H contacts to the Hirshfeld surface, showing two narrow spikes, provides evidence for the presence of inter­molecular C—H⋯O inter­actions in compound (I). Furthermore, the significant C—H⋯π inter­actions in both (I) and (II) are indicated by the wings at *d_e_* + *d_i_* 2.7 Å.

## Database survey   

A survey of Cambridge Structural Database (CSD, Version 5.38, last update November 2016; Groom *et al.*, 2016 ▸) revealed four compounds having an anthracene-ketone substit­uent on the chalcone, *i.e.* anthracen-9-yl styryl ketone and 9,10-anthryl bis­(styryl ketone) (Harlow *et al.*, 1975 ▸), (2*E*)-1-(anthracen-9-yl)-3-[4-(propan-2-yl)phen­yl]prop-2-en-1-one (Girisha *et al.*, 2016 ▸) and (*E*)-1-(anthracen-9-yl)-3-(2-chloro-6-fluoro­phen­yl)prop-2-en-1-one (Abdullah *et al.*, 2016 ▸). Zainuri *et al.* (2018 ▸) reported the structure of the bis-substituted anthracene chalcone, (*E*)-1,3-bis­(anthracen-9-yl)prop-2-en-1-one. Others related compounds include 1-(anthracen-9-yl)-2-meth­ylprop-2-en-1-one (Agrahari *et al.*, 2015 ▸) and 9-anthroylacetone (Cicogna *et al.*, 2004 ▸).

## Synthesis and crystallization   

A mixture of 9-acetyl­anthracene (0.5 mmol) and 2-napthaldehyde or 1-pyrenecarboxaldehyde (0.5 mmol) for compounds (I) and (II), respectively, was dissolved in methanol (20 ml). A catalytic amount of NaOH (5 ml, 20%) was added to the solution dropwise with vigorous stirring. The reaction mixture was stirred for about 6 h at room temperature. After stirring, the contents of the flask were poured into ice-cold water (50 ml). The resultant crude products were filtered, washed successively with distilled water and recrystallized from acetone to get the corresponding chalcones. Single crystals of (I) and (II) suitable for X-ray diffraction analysis were obtained by slow evaporation of an acetone solution.

## Refinement   

Crystal data collection and structure refinement details are summarized in Table 3 ▸. All H atoms were positioned geometrically [C—H = 0.95 Å in (I) and 0.93 Å in (II)] and refined using riding model with *U*_iso_(H) = 1.2*U*_eq_(C).

## Supplementary Material

Crystal structure: contains datablock(s) I, II. DOI: 10.1107/S2056989018005467/rz5230sup1.cif

Structure factors: contains datablock(s) I. DOI: 10.1107/S2056989018005467/rz5230Isup2.hkl

Structure factors: contains datablock(s) II. DOI: 10.1107/S2056989018005467/rz5230IIsup3.hkl

Supporting information file. DOI: 10.1107/S2056989018005467/rz5230Isup4.cml

Supporting information file. DOI: 10.1107/S2056989018005467/rz5230IIsup5.cml

CCDC references: 1817219, 1817253

Additional supporting information:  crystallographic information; 3D view; checkCIF report

## Figures and Tables

**Table 1:** Hydrogen-bond geometry (Å, °) for (I) *Cg*4 is the centroid of the C18–C20/C25–C27 ring

*D*—H⋯*A*	*D*—H	H⋯*A*	*D*⋯*A*	*D*—H⋯*A*
C27—H27*A*⋯O1^i^	0.95	2.36	3.2721 (14)	161
C10—H10*A*⋯*Cg*4^ii^	0.95	2.85	3.6610 (14)	142

Symmetry codes: (i) 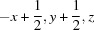; (ii) 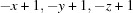.

**Table 2:** Hydrogen-bond geometry (Å, °) for (II) *Cg*3, *Cg*4 and *Cg*5 are the centroids of the C18–C13, C21–C24/C32/C33 and C18–C21/C31/C32 rings

*D*—H⋯*A*	*D*—H	H⋯*A*	*D*⋯*A*	*D*—H⋯*A*
C25—H25*A*⋯*Cg*3^i^	0.93	2.82	3.7220 (3)	164
C26—H26*A*⋯*Cg*5^ii^	0.93	2.81	3.6050 (3)	144
C27—H27*A*⋯*Cg*4^ii^	0.93	2.95	3.6520 (3)	134

Symmetry codes: (i) 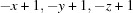; (ii) 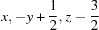.

**Table 3:** Experimental details

	(I)	(II)
Crystal data		
Chemical formula	C_27_H_18_O	C_33_H_20_O
*M*_r_	358.41	432.49
Crystal system, space group	Orthorhombic, *P* *b* *c* *a*	Monoclinic, *P*2_1_/*c*
Temperature (K)	100	296
*a*, *b*, *c* (Å)	13.2129 (10), 11.1224 (8), 25.1604 (19)	17.118 (5), 12.310 (4), 11.152 (3)
α, β, γ (°)	90, 90, 90	90, 107.929 (5), 90
*V* (Å^3^)	3697.6 (5)	2235.8 (12)
*Z*	8	4
Radiation type	Mo *K*α	Mo *K*α
μ (mm^−1^)	0.08	0.08
Crystal size (mm)	0.87 × 0.43 × 0.20	0.66 × 0.66 × 0.26

Data collection		
Diffractometer	Bruker SMART APEXII Duo CCD area-detector	Bruker SMART APEXII Duo CCD area-detector
Absorption correction	Multi-scan (*SADABS*; Bruker, 2009 ▸)	Multi-scan *SADABS* 2014/5
*T*_min_, *T*_max_	0.502, 0.746	0.700, 0.927
No. of measured, independent and observed [*I* > 2σ(*I*)] reflections	96576, 4581, 3919	39809, 4394, 2739
*R*_int_	0.067	0.048
(sin θ/λ)_max_ (Å^−1^)	0.667	0.617

Refinement		
*R*[*F* ^2^ > 2σ(*F* ^2^)], *wR*(*F* ^2^), *S*	0.043, 0.114, 1.04	0.046, 0.137, 1.09
No. of reflections	4581	4394
No. of parameters	253	307
H-atom treatment	H-atom parameters constrained	H-atom parameters constrained
Δρ_max_, Δρ_min_ (e Å^−3^)	0.26, −0.24	0.15, −0.14

Computer programs: *APEX2* and *SAINT* (Bruker, 2009 ▸), *SHELXL2013* and *SHELXL2014* (Sheldrick, 2015 ▸), *SHELXTL* (Sheldrick, 2008 ▸) and *PLATON* (Spek, 2009 ▸).

## supplementary crystallographic information

### 1-(Anthracen-9-yl)-3-(naphthalen-2-yl)prop-2-en-1-one (I) Crystal data

**Table:**

C_27_H_18_O	*D*_x_ = 1.288 Mg m^−^^3^
*M**_r_* = 358.41	Mo *K*α radiation, λ = 0.71073 Å
Orthorhombic, *P**b**c**a*	Cell parameters from 9963 reflections
*a* = 13.2129 (10) Å	θ = 2.2–28.1°
*b* = 11.1224 (8) Å	µ = 0.08 mm^−^^1^
*c* = 25.1604 (19) Å	*T* = 100 K
*V* = 3697.6 (5) Å^3^	Plate, yellow
*Z* = 8	0.87 × 0.43 × 0.20 mm
*F*(000) = 1504	

### 1-(Anthracen-9-yl)-3-(naphthalen-2-yl)prop-2-en-1-one (I) Data collection

**Table:**

Bruker SMART APEXII Duo CCD area-detector diffractometer	3919 reflections with *I* > 2σ(*I*)
Radiation source: fine-focus sealed tube	*R*_int_ = 0.067
φ and ω scans	θ_max_ = 28.3°, θ_min_ = 1.6°
Absorption correction: multi-scan (*SADABS*; Bruker, 2009)	*h* = −17→17
*T*_min_ = 0.502, *T*_max_ = 0.746	*k* = −14→14
96576 measured reflections	*l* = −33→33
4581 independent reflections	

### 1-(Anthracen-9-yl)-3-(naphthalen-2-yl)prop-2-en-1-one (I) Refinement

**Table:**

Refinement on *F*^2^	0 restraints
Least-squares matrix: full	Hydrogen site location: inferred from neighbouring sites
*R*[*F*^2^ > 2σ(*F*^2^)] = 0.043	H-atom parameters constrained
*wR*(*F*^2^) = 0.114	*w* = 1/[σ^2^(*F*_o_^2^) + (0.0514*P*)^2^ + 1.843*P*] where *P* = (*F*_o_^2^ + 2*F*_c_^2^)/3
*S* = 1.04	(Δ/σ)_max_ < 0.001
4581 reflections	Δρ_max_ = 0.26 e Å^−^^3^
253 parameters	Δρ_min_ = −0.24 e Å^−^^3^

### 1-(Anthracen-9-yl)-3-(naphthalen-2-yl)prop-2-en-1-one (I) Special details

**Table:**

Geometry. All esds (except the esd in the dihedral angle between two l.s. planes) are estimated using the full covariance matrix. The cell esds are taken into account individually in the estimation of esds in distances, angles and torsion angles; correlations between esds in cell parameters are only used when they are defined by crystal symmetry. An approximate (isotropic) treatment of cell esds is used for estimating esds involving l.s. planes.

### 1-(Anthracen-9-yl)-3-(naphthalen-2-yl)prop-2-en-1-one (I) Fractional atomic coordinates and isotropic or equivalent isotropic displacement parameters (Å^2^)

**Table:**

	*x*	*y*	*z*	*U*_iso_*/*U*_eq_	
O1	0.15754 (7)	0.24179 (8)	0.41888 (4)	0.0269 (2)	
C1	0.08186 (8)	0.50963 (10)	0.42203 (4)	0.0177 (2)	
C2	0.03476 (9)	0.47889 (11)	0.37257 (4)	0.0211 (2)	
H2A	0.0611	0.4144	0.3521	0.025*	
C3	−0.04727 (9)	0.54078 (11)	0.35452 (5)	0.0248 (3)	
H3A	−0.0768	0.5196	0.3214	0.030*	
C4	−0.08930 (9)	0.63697 (11)	0.38471 (5)	0.0253 (3)	
H4A	−0.1471	0.6787	0.3719	0.030*	
C5	−0.04678 (9)	0.66920 (10)	0.43191 (5)	0.0227 (2)	
H5A	−0.0755	0.7333	0.4518	0.027*	
C6	0.04037 (8)	0.60806 (10)	0.45192 (4)	0.0185 (2)	
C7	0.08565 (9)	0.64129 (10)	0.49988 (4)	0.0196 (2)	
H7A	0.0565	0.7043	0.5203	0.024*	
C8	0.17270 (9)	0.58413 (10)	0.51852 (4)	0.0186 (2)	
C9	0.22208 (9)	0.62058 (11)	0.56649 (4)	0.0226 (2)	
H9A	0.1955	0.6861	0.5863	0.027*	
C10	0.30638 (10)	0.56309 (11)	0.58417 (5)	0.0251 (3)	
H10A	0.3381	0.5886	0.6161	0.030*	
C11	0.34727 (10)	0.46500 (11)	0.55501 (5)	0.0258 (3)	
H11A	0.4064	0.4256	0.5676	0.031*	
C12	0.30260 (9)	0.42693 (11)	0.50918 (5)	0.0223 (2)	
H12A	0.3307	0.3608	0.4903	0.027*	
C13	0.21408 (8)	0.48513 (10)	0.48908 (4)	0.0179 (2)	
C14	0.16655 (8)	0.44797 (10)	0.44189 (4)	0.0172 (2)	
C15	0.20387 (9)	0.33672 (10)	0.41364 (4)	0.0189 (2)	
C16	0.29376 (9)	0.34358 (11)	0.37952 (4)	0.0211 (2)	
H16A	0.3175	0.2724	0.3628	0.025*	
C17	0.34341 (8)	0.44712 (11)	0.37123 (4)	0.0195 (2)	
H17A	0.3184	0.5158	0.3894	0.023*	
C18	0.43188 (8)	0.46569 (10)	0.33722 (4)	0.0195 (2)	
C19	0.47087 (9)	0.37481 (11)	0.30286 (4)	0.0217 (2)	
H19A	0.4391	0.2982	0.3015	0.026*	
C20	0.55373 (9)	0.39751 (11)	0.27192 (4)	0.0223 (2)	
H20A	0.5775	0.3369	0.2485	0.027*	
C21	0.60512 (9)	0.50964 (11)	0.27401 (4)	0.0202 (2)	
C22	0.69383 (9)	0.53418 (11)	0.24427 (5)	0.0240 (3)	
H22A	0.7192	0.4754	0.2204	0.029*	
C23	0.74340 (10)	0.64171 (12)	0.24957 (5)	0.0268 (3)	
H23A	0.8035	0.6561	0.2298	0.032*	
C24	0.70585 (9)	0.73139 (12)	0.28419 (5)	0.0255 (3)	
H24A	0.7410	0.8055	0.2878	0.031*	
C25	0.61895 (9)	0.71144 (11)	0.31242 (4)	0.0222 (2)	
H25A	0.5932	0.7728	0.3349	0.027*	
C26	0.56693 (8)	0.60044 (10)	0.30848 (4)	0.0194 (2)	
C27	0.47889 (8)	0.57612 (10)	0.33878 (4)	0.0193 (2)	
H27A	0.4516	0.6376	0.3607	0.023*	

### 1-(Anthracen-9-yl)-3-(naphthalen-2-yl)prop-2-en-1-one (I) Atomic displacement parameters (Å^2^)

**Table:**

	*U*^11^	*U*^22^	*U*^33^	*U*^12^	*U*^13^	*U*^23^
O1	0.0302 (5)	0.0210 (4)	0.0294 (5)	−0.0044 (3)	−0.0006 (4)	−0.0043 (3)
C1	0.0196 (5)	0.0191 (5)	0.0143 (5)	−0.0023 (4)	0.0018 (4)	0.0014 (4)
C2	0.0232 (5)	0.0243 (6)	0.0157 (5)	−0.0014 (4)	−0.0002 (4)	−0.0006 (4)
C3	0.0256 (6)	0.0299 (6)	0.0191 (5)	−0.0017 (5)	−0.0036 (4)	0.0015 (5)
C4	0.0225 (6)	0.0266 (6)	0.0269 (6)	0.0021 (5)	−0.0018 (5)	0.0052 (5)
C5	0.0233 (6)	0.0197 (5)	0.0251 (6)	0.0008 (4)	0.0031 (4)	0.0025 (4)
C6	0.0196 (5)	0.0182 (5)	0.0176 (5)	−0.0022 (4)	0.0030 (4)	0.0022 (4)
C7	0.0240 (5)	0.0171 (5)	0.0177 (5)	−0.0021 (4)	0.0045 (4)	−0.0009 (4)
C8	0.0239 (5)	0.0178 (5)	0.0141 (5)	−0.0035 (4)	0.0021 (4)	0.0004 (4)
C9	0.0319 (6)	0.0204 (5)	0.0154 (5)	−0.0033 (5)	0.0017 (4)	−0.0022 (4)
C10	0.0357 (7)	0.0247 (6)	0.0150 (5)	−0.0058 (5)	−0.0050 (5)	−0.0009 (4)
C11	0.0312 (6)	0.0253 (6)	0.0209 (6)	0.0015 (5)	−0.0078 (5)	0.0007 (5)
C12	0.0278 (6)	0.0203 (5)	0.0188 (5)	0.0020 (4)	−0.0032 (4)	−0.0009 (4)
C13	0.0220 (5)	0.0174 (5)	0.0142 (5)	−0.0024 (4)	0.0002 (4)	0.0003 (4)
C14	0.0205 (5)	0.0170 (5)	0.0141 (5)	−0.0019 (4)	0.0013 (4)	0.0004 (4)
C15	0.0221 (5)	0.0201 (5)	0.0146 (5)	0.0006 (4)	−0.0045 (4)	−0.0020 (4)
C16	0.0243 (6)	0.0226 (5)	0.0163 (5)	0.0037 (4)	−0.0024 (4)	−0.0039 (4)
C17	0.0211 (5)	0.0240 (5)	0.0134 (5)	0.0042 (4)	−0.0028 (4)	−0.0019 (4)
C18	0.0198 (5)	0.0249 (6)	0.0137 (5)	0.0036 (4)	−0.0028 (4)	−0.0011 (4)
C19	0.0243 (5)	0.0244 (6)	0.0165 (5)	0.0016 (4)	−0.0028 (4)	−0.0037 (4)
C20	0.0258 (6)	0.0266 (6)	0.0144 (5)	0.0051 (5)	−0.0012 (4)	−0.0046 (4)
C21	0.0223 (5)	0.0266 (6)	0.0118 (5)	0.0037 (4)	−0.0025 (4)	−0.0002 (4)
C22	0.0260 (6)	0.0307 (6)	0.0154 (5)	0.0046 (5)	0.0022 (4)	−0.0013 (4)
C23	0.0250 (6)	0.0349 (6)	0.0205 (5)	0.0015 (5)	0.0030 (5)	0.0037 (5)
C24	0.0267 (6)	0.0274 (6)	0.0223 (6)	−0.0008 (5)	−0.0015 (5)	0.0036 (5)
C25	0.0258 (6)	0.0235 (6)	0.0174 (5)	0.0034 (4)	−0.0022 (4)	−0.0003 (4)
C26	0.0215 (5)	0.0248 (6)	0.0120 (5)	0.0044 (4)	−0.0030 (4)	0.0006 (4)
C27	0.0209 (5)	0.0239 (5)	0.0132 (5)	0.0050 (4)	−0.0020 (4)	−0.0015 (4)

### 1-(Anthracen-9-yl)-3-(naphthalen-2-yl)prop-2-en-1-one (I) Geometric parameters (Å, º)

**Table:**

O1—C15	1.2275 (14)	C13—C14	1.4052 (15)
C1—C14	1.4044 (15)	C14—C15	1.5097 (15)
C1—C2	1.4328 (15)	C15—C16	1.4675 (16)
C1—C6	1.4370 (15)	C16—C17	1.3416 (17)
C2—C3	1.3619 (17)	C16—H16A	0.9500
C2—H2A	0.9500	C17—C18	1.4634 (16)
C3—C4	1.4247 (18)	C17—H17A	0.9500
C3—H3A	0.9500	C18—C27	1.3769 (16)
C4—C5	1.3618 (17)	C18—C19	1.4265 (15)
C4—H4A	0.9500	C19—C20	1.3668 (17)
C5—C6	1.4290 (16)	C19—H19A	0.9500
C5—H5A	0.9500	C20—C21	1.4210 (17)
C6—C7	1.3965 (16)	C20—H20A	0.9500
C7—C8	1.3954 (16)	C21—C22	1.4171 (16)
C7—H7A	0.9500	C21—C26	1.4237 (15)
C8—C9	1.4306 (15)	C22—C23	1.3701 (18)
C8—C13	1.4353 (15)	C22—H22A	0.9500
C9—C10	1.3593 (18)	C23—C24	1.4142 (18)
C9—H9A	0.9500	C23—H23A	0.9500
C10—C11	1.4214 (17)	C24—C25	1.3682 (17)
C10—H10A	0.9500	C24—H24A	0.9500
C11—C12	1.3629 (16)	C25—C26	1.4165 (17)
C11—H11A	0.9500	C25—H25A	0.9500
C12—C13	1.4292 (16)	C26—C27	1.4168 (16)
C12—H12A	0.9500	C27—H27A	0.9500
			
C14—C1—C2	122.59 (10)	C13—C14—C15	119.53 (10)
C14—C1—C6	119.32 (10)	O1—C15—C16	120.73 (10)
C2—C1—C6	118.08 (10)	O1—C15—C14	119.45 (10)
C3—C2—C1	120.97 (11)	C16—C15—C14	119.81 (10)
C3—C2—H2A	119.5	C17—C16—C15	122.09 (10)
C1—C2—H2A	119.5	C17—C16—H16A	119.0
C2—C3—C4	120.80 (11)	C15—C16—H16A	119.0
C2—C3—H3A	119.6	C16—C17—C18	127.06 (11)
C4—C3—H3A	119.6	C16—C17—H17A	116.5
C5—C4—C3	120.12 (11)	C18—C17—H17A	116.5
C5—C4—H4A	119.9	C27—C18—C19	119.10 (10)
C3—C4—H4A	119.9	C27—C18—C17	118.01 (10)
C4—C5—C6	120.96 (11)	C19—C18—C17	122.88 (11)
C4—C5—H5A	119.5	C20—C19—C18	120.24 (11)
C6—C5—H5A	119.5	C20—C19—H19A	119.9
C7—C6—C5	121.58 (10)	C18—C19—H19A	119.9
C7—C6—C1	119.37 (10)	C19—C20—C21	121.59 (11)
C5—C6—C1	119.05 (10)	C19—C20—H20A	119.2
C8—C7—C6	121.54 (10)	C21—C20—H20A	119.2
C8—C7—H7A	119.2	C22—C21—C20	123.01 (11)
C6—C7—H7A	119.2	C22—C21—C26	118.57 (11)
C7—C8—C9	122.03 (10)	C20—C21—C26	118.41 (10)
C7—C8—C13	119.34 (10)	C23—C22—C21	120.81 (11)
C9—C8—C13	118.63 (10)	C23—C22—H22A	119.6
C10—C9—C8	121.10 (11)	C21—C22—H22A	119.6
C10—C9—H9A	119.5	C22—C23—C24	120.53 (12)
C8—C9—H9A	119.5	C22—C23—H23A	119.7
C9—C10—C11	120.24 (11)	C24—C23—H23A	119.7
C9—C10—H10A	119.9	C25—C24—C23	119.98 (12)
C11—C10—H10A	119.9	C25—C24—H24A	120.0
C12—C11—C10	120.70 (11)	C23—C24—H24A	120.0
C12—C11—H11A	119.6	C24—C25—C26	120.82 (11)
C10—C11—H11A	119.6	C24—C25—H25A	119.6
C11—C12—C13	120.87 (11)	C26—C25—H25A	119.6
C11—C12—H12A	119.6	C25—C26—C27	121.82 (10)
C13—C12—H12A	119.6	C25—C26—C21	119.27 (10)
C14—C13—C12	122.10 (10)	C27—C26—C21	118.90 (11)
C14—C13—C8	119.44 (10)	C18—C27—C26	121.70 (10)
C12—C13—C8	118.46 (10)	C18—C27—H27A	119.2
C1—C14—C13	120.87 (10)	C26—C27—H27A	119.2
C1—C14—C15	119.53 (9)		
			
C14—C1—C2—C3	−179.94 (11)	C12—C13—C14—C15	5.41 (16)
C6—C1—C2—C3	−0.31 (16)	C8—C13—C14—C15	−174.20 (10)
C1—C2—C3—C4	−0.79 (18)	C1—C14—C15—O1	−76.15 (14)
C2—C3—C4—C5	0.86 (18)	C13—C14—C15—O1	100.87 (13)
C3—C4—C5—C6	0.21 (18)	C1—C14—C15—C16	102.72 (12)
C4—C5—C6—C7	179.05 (11)	C13—C14—C15—C16	−80.26 (13)
C4—C5—C6—C1	−1.30 (17)	O1—C15—C16—C17	177.11 (11)
C14—C1—C6—C7	0.64 (16)	C14—C15—C16—C17	−1.74 (16)
C2—C1—C6—C7	−179.01 (10)	C15—C16—C17—C18	−178.36 (10)
C14—C1—C6—C5	−179.02 (10)	C16—C17—C18—C27	−172.68 (11)
C2—C1—C6—C5	1.33 (15)	C16—C17—C18—C19	7.35 (18)
C5—C6—C7—C8	−177.86 (10)	C27—C18—C19—C20	−0.07 (16)
C1—C6—C7—C8	2.49 (16)	C17—C18—C19—C20	179.89 (10)
C6—C7—C8—C9	177.43 (10)	C18—C19—C20—C21	1.88 (17)
C6—C7—C8—C13	−2.98 (16)	C19—C20—C21—C22	177.08 (11)
C7—C8—C9—C10	179.50 (11)	C19—C20—C21—C26	−1.46 (16)
C13—C8—C9—C10	−0.10 (17)	C20—C21—C22—C23	−176.84 (11)
C8—C9—C10—C11	0.05 (18)	C26—C21—C22—C23	1.70 (17)
C9—C10—C11—C12	−0.25 (19)	C21—C22—C23—C24	−1.17 (18)
C10—C11—C12—C13	0.48 (19)	C22—C23—C24—C25	−0.48 (18)
C11—C12—C13—C14	179.87 (11)	C23—C24—C25—C26	1.55 (17)
C11—C12—C13—C8	−0.51 (17)	C24—C25—C26—C27	177.67 (11)
C7—C8—C13—C14	0.33 (16)	C24—C25—C26—C21	−0.98 (16)
C9—C8—C13—C14	179.94 (10)	C22—C21—C26—C25	−0.63 (16)
C7—C8—C13—C12	−179.29 (10)	C20—C21—C26—C25	177.98 (10)
C9—C8—C13—C12	0.32 (16)	C22—C21—C26—C27	−179.32 (10)
C2—C1—C14—C13	176.37 (10)	C20—C21—C26—C27	−0.72 (15)
C6—C1—C14—C13	−3.26 (16)	C19—C18—C27—C26	−2.15 (16)
C2—C1—C14—C15	−6.65 (16)	C17—C18—C27—C26	177.88 (10)
C6—C1—C14—C15	173.72 (10)	C25—C26—C27—C18	−176.12 (10)
C12—C13—C14—C1	−177.61 (10)	C21—C26—C27—C18	2.54 (16)
C8—C13—C14—C1	2.78 (16)		

### 1-(Anthracen-9-yl)-3-(naphthalen-2-yl)prop-2-en-1-one (I) Hydrogen-bond geometry (Å, º)

Cg4 is the centroid of the C18–C20/C25–C27 ring

**Table:**

*D*—H···*A*	*D*—H	H···*A*	*D*···*A*	*D*—H···*A*
C27—H27*A*···O1^i^	0.95	2.36	3.2721 (14)	161
C10—H10*A*···*Cg*4^ii^	0.95	2.85	3.6610 (14)	142

Symmetry codes: (i) −*x*+1/2, *y*+1/2, *z*; (ii) −*x*+1, −*y*+1, −*z*+1.

### 1-(Anthracen-9-yl)-3-(pyren-1-yl)prop-2-en-1-one (II) Crystal data

**Table:**

C_33_H_20_O	*F*(000) = 904
*M**_r_* = 432.49	*D*_x_ = 1.285 Mg m^−^^3^
Monoclinic, *P*2_1_/*c*	Mo *K*α radiation, λ = 0.71073 Å
*a* = 17.118 (5) Å	Cell parameters from 5604 reflections
*b* = 12.310 (4) Å	θ = 2.5–23.7°
*c* = 11.152 (3) Å	µ = 0.08 mm^−^^1^
β = 107.929 (5)°	*T* = 296 K
*V* = 2235.8 (12) Å^3^	Plate, yellow
*Z* = 4	0.66 × 0.66 × 0.26 mm

### 1-(Anthracen-9-yl)-3-(pyren-1-yl)prop-2-en-1-one (II) Data collection

**Table:**

Bruker SMART APEXII Duo CCD area-detector diffractometer	2739 reflections with *I* > 2σ(*I*)
Radiation source: fine-focus sealed tube	*R*_int_ = 0.048
φ and ω scans	θ_max_ = 26.0°, θ_min_ = 1.3°
Absorption correction: multi-scan SADABS 2014/5	*h* = −21→21
*T*_min_ = 0.700, *T*_max_ = 0.927	*k* = −15→15
39809 measured reflections	*l* = −13→13
4394 independent reflections	

### 1-(Anthracen-9-yl)-3-(pyren-1-yl)prop-2-en-1-one (II) Refinement

**Table:**

Refinement on *F*^2^	0 restraints
Least-squares matrix: full	Hydrogen site location: inferred from neighbouring sites
*R*[*F*^2^ > 2σ(*F*^2^)] = 0.046	H-atom parameters constrained
*wR*(*F*^2^) = 0.137	*w* = 1/[σ^2^(*F*_o_^2^) + (0.0446*P*)^2^ + 0.5972*P*] where *P* = (*F*_o_^2^ + 2*F*_c_^2^)/3
*S* = 1.09	(Δ/σ)_max_ < 0.001
4394 reflections	Δρ_max_ = 0.15 e Å^−^^3^
307 parameters	Δρ_min_ = −0.14 e Å^−^^3^

### 1-(Anthracen-9-yl)-3-(pyren-1-yl)prop-2-en-1-one (II) Special details

**Table:**

Experimental. The following wavelength and cell were deduced by SADABS from the direction cosines etc. They are given here for emergency use only: CELL 0.71104 11.238 12.388 17.234 90.027 107.859 90.073
Geometry. All esds (except the esd in the dihedral angle between two l.s. planes) are estimated using the full covariance matrix. The cell esds are taken into account individually in the estimation of esds in distances, angles and torsion angles; correlations between esds in cell parameters are only used when they are defined by crystal symmetry. An approximate (isotropic) treatment of cell esds is used for estimating esds involving l.s. planes.

### 1-(Anthracen-9-yl)-3-(pyren-1-yl)prop-2-en-1-one (II) Fractional atomic coordinates and isotropic or equivalent isotropic displacement parameters (Å^2^)

**Table:**

	*x*	*y*	*z*	*U*_iso_*/*U*_eq_	
O1	0.17677 (10)	0.46286 (15)	0.87794 (14)	0.0885 (5)	
C1	0.14507 (12)	0.62079 (18)	0.6345 (2)	0.0624 (5)	
C2	0.17465 (15)	0.70424 (19)	0.7246 (2)	0.0779 (7)	
H2A	0.2030	0.6861	0.8076	0.093*	
C3	0.16206 (18)	0.8103 (2)	0.6912 (3)	0.0982 (9)	
H3A	0.1814	0.8642	0.7515	0.118*	
C4	0.12019 (19)	0.8391 (3)	0.5668 (4)	0.1063 (10)	
H4A	0.1126	0.9122	0.5449	0.128*	
C5	0.09075 (17)	0.7625 (3)	0.4782 (3)	0.0944 (8)	
H5A	0.0626	0.7833	0.3961	0.113*	
C6	0.10194 (13)	0.6505 (2)	0.5082 (2)	0.0713 (6)	
C7	0.07398 (13)	0.5692 (2)	0.4191 (2)	0.0783 (7)	
H7A	0.0454	0.5887	0.3367	0.094*	
C8	0.08713 (12)	0.4605 (2)	0.4484 (2)	0.0690 (6)	
C9	0.06052 (14)	0.3764 (3)	0.3561 (2)	0.0871 (8)	
H9A	0.0325	0.3946	0.2730	0.105*	
C10	0.07563 (16)	0.2710 (3)	0.3882 (3)	0.0925 (8)	
H10A	0.0582	0.2174	0.3270	0.111*	
C11	0.11715 (15)	0.2417 (2)	0.5122 (3)	0.0832 (7)	
H11A	0.1266	0.1687	0.5329	0.100*	
C12	0.14376 (13)	0.31760 (19)	0.6026 (2)	0.0698 (6)	
H12A	0.1715	0.2962	0.6847	0.084*	
C13	0.13005 (11)	0.42991 (18)	0.57442 (19)	0.0593 (5)	
C14	0.15845 (12)	0.51091 (17)	0.66534 (18)	0.0574 (5)	
C15	0.20900 (13)	0.47913 (16)	0.79620 (19)	0.0625 (5)	
C16	0.29722 (13)	0.47072 (17)	0.82128 (18)	0.0650 (6)	
H16A	0.3292	0.4442	0.8990	0.078*	
C17	0.33365 (12)	0.49963 (16)	0.73682 (18)	0.0573 (5)	
H17A	0.2989	0.5221	0.6590	0.069*	
C18	0.42074 (12)	0.50067 (15)	0.75046 (16)	0.0531 (5)	
C19	0.47586 (13)	0.44278 (16)	0.84749 (18)	0.0631 (5)	
H19A	0.4561	0.4033	0.9031	0.076*	
C20	0.55833 (13)	0.44252 (17)	0.86316 (18)	0.0649 (5)	
H20A	0.5933	0.4026	0.9287	0.078*	
C21	0.59063 (12)	0.50066 (16)	0.78327 (17)	0.0551 (5)	
C22	0.67696 (13)	0.50448 (18)	0.7997 (2)	0.0680 (6)	
H22A	0.7130	0.4666	0.8661	0.082*	
C23	0.70663 (13)	0.56110 (19)	0.7218 (2)	0.0705 (6)	
H23A	0.7630	0.5620	0.7354	0.085*	
C24	0.65461 (13)	0.62047 (16)	0.61809 (19)	0.0594 (5)	
C25	0.68439 (15)	0.67865 (19)	0.5348 (2)	0.0749 (6)	
H25A	0.7406	0.6805	0.5469	0.090*	
C26	0.63215 (17)	0.73339 (18)	0.4350 (2)	0.0788 (7)	
H26A	0.6533	0.7712	0.3798	0.095*	
C27	0.54903 (15)	0.73297 (16)	0.4157 (2)	0.0691 (6)	
H27A	0.5145	0.7708	0.3478	0.083*	
C28	0.51601 (13)	0.67643 (15)	0.49669 (17)	0.0549 (5)	
C29	0.43069 (13)	0.67432 (15)	0.48102 (18)	0.0591 (5)	
H29A	0.3951	0.7117	0.4137	0.071*	
C30	0.39964 (12)	0.61986 (15)	0.56071 (17)	0.0557 (5)	
H30A	0.3433	0.6217	0.5478	0.067*	
C31	0.45105 (11)	0.55896 (14)	0.66509 (15)	0.0475 (4)	
C32	0.53673 (11)	0.55946 (14)	0.68243 (16)	0.0486 (4)	
C33	0.56895 (11)	0.61898 (14)	0.59947 (16)	0.0505 (4)	

### 1-(Anthracen-9-yl)-3-(pyren-1-yl)prop-2-en-1-one (II) Atomic displacement parameters (Å^2^)

**Table:**

	*U*^11^	*U*^22^	*U*^33^	*U*^12^	*U*^13^	*U*^23^
O1	0.0916 (12)	0.1139 (14)	0.0734 (10)	−0.0170 (10)	0.0451 (9)	0.0037 (9)
C1	0.0527 (12)	0.0709 (14)	0.0726 (13)	−0.0068 (11)	0.0327 (10)	−0.0003 (11)
C2	0.0823 (17)	0.0696 (15)	0.0930 (17)	−0.0158 (13)	0.0435 (13)	−0.0055 (13)
C3	0.110 (2)	0.0706 (18)	0.135 (3)	−0.0190 (15)	0.069 (2)	−0.0066 (17)
C4	0.106 (2)	0.0743 (19)	0.160 (3)	0.0064 (17)	0.072 (2)	0.030 (2)
C5	0.0829 (19)	0.096 (2)	0.114 (2)	0.0105 (16)	0.0439 (16)	0.0293 (18)
C6	0.0562 (13)	0.0819 (17)	0.0833 (15)	0.0032 (12)	0.0324 (12)	0.0153 (13)
C7	0.0541 (14)	0.111 (2)	0.0690 (14)	0.0022 (13)	0.0172 (11)	0.0113 (14)
C8	0.0466 (12)	0.0953 (18)	0.0670 (13)	−0.0059 (12)	0.0202 (10)	−0.0061 (12)
C9	0.0581 (15)	0.131 (2)	0.0695 (15)	−0.0141 (16)	0.0161 (11)	−0.0231 (16)
C10	0.0708 (17)	0.112 (2)	0.100 (2)	−0.0216 (16)	0.0331 (15)	−0.0421 (18)
C11	0.0720 (16)	0.0844 (17)	0.1006 (19)	−0.0136 (13)	0.0374 (15)	−0.0248 (15)
C12	0.0616 (14)	0.0754 (15)	0.0781 (14)	−0.0078 (11)	0.0300 (11)	−0.0104 (12)
C13	0.0446 (11)	0.0735 (14)	0.0654 (12)	−0.0071 (10)	0.0255 (9)	−0.0064 (10)
C14	0.0495 (11)	0.0668 (13)	0.0629 (12)	−0.0077 (10)	0.0276 (9)	−0.0016 (10)
C15	0.0711 (14)	0.0635 (13)	0.0598 (12)	−0.0152 (11)	0.0303 (11)	−0.0039 (10)
C16	0.0643 (14)	0.0771 (14)	0.0522 (11)	−0.0100 (11)	0.0159 (10)	0.0015 (10)
C17	0.0571 (12)	0.0611 (12)	0.0532 (11)	−0.0047 (10)	0.0160 (9)	0.0007 (9)
C18	0.0548 (12)	0.0535 (11)	0.0490 (10)	−0.0017 (9)	0.0129 (9)	−0.0009 (8)
C19	0.0672 (14)	0.0651 (13)	0.0554 (11)	−0.0044 (11)	0.0167 (10)	0.0087 (10)
C20	0.0639 (14)	0.0671 (13)	0.0565 (11)	0.0091 (11)	0.0081 (10)	0.0110 (10)
C21	0.0534 (12)	0.0560 (11)	0.0520 (10)	0.0032 (9)	0.0104 (9)	−0.0042 (9)
C22	0.0557 (13)	0.0738 (14)	0.0685 (13)	0.0099 (11)	0.0104 (10)	−0.0021 (11)
C23	0.0509 (12)	0.0775 (15)	0.0825 (15)	0.0010 (11)	0.0197 (11)	−0.0111 (12)
C24	0.0591 (13)	0.0544 (12)	0.0693 (12)	−0.0039 (10)	0.0264 (10)	−0.0130 (10)
C25	0.0730 (15)	0.0687 (14)	0.0951 (17)	−0.0120 (12)	0.0434 (14)	−0.0130 (13)
C26	0.099 (2)	0.0656 (15)	0.0888 (17)	−0.0090 (13)	0.0529 (15)	0.0009 (12)
C27	0.0876 (17)	0.0551 (12)	0.0725 (14)	−0.0002 (12)	0.0365 (12)	0.0043 (10)
C28	0.0675 (14)	0.0437 (10)	0.0567 (11)	−0.0014 (9)	0.0241 (10)	−0.0035 (8)
C29	0.0682 (14)	0.0512 (11)	0.0558 (11)	0.0073 (10)	0.0159 (10)	0.0065 (9)
C30	0.0521 (11)	0.0541 (11)	0.0592 (11)	0.0040 (9)	0.0145 (9)	0.0010 (9)
C31	0.0522 (11)	0.0435 (10)	0.0455 (9)	0.0000 (8)	0.0131 (8)	−0.0034 (8)
C32	0.0519 (11)	0.0445 (10)	0.0483 (10)	0.0008 (8)	0.0136 (8)	−0.0062 (8)
C33	0.0585 (12)	0.0422 (10)	0.0541 (10)	−0.0023 (9)	0.0222 (9)	−0.0090 (8)

### 1-(Anthracen-9-yl)-3-(pyren-1-yl)prop-2-en-1-one (II) Geometric parameters (Å, º)

**Table:**

O1—C15	1.219 (2)	C17—C18	1.451 (3)
C1—C14	1.397 (3)	C17—H17A	0.9300
C1—C2	1.417 (3)	C18—C19	1.393 (3)
C1—C6	1.423 (3)	C18—C31	1.412 (2)
C2—C3	1.356 (3)	C19—C20	1.368 (3)
C2—H2A	0.9300	C19—H19A	0.9300
C3—C4	1.397 (4)	C20—C21	1.384 (3)
C3—H3A	0.9300	C20—H20A	0.9300
C4—C5	1.346 (4)	C21—C32	1.415 (2)
C4—H4A	0.9300	C21—C22	1.433 (3)
C5—C6	1.417 (3)	C22—C23	1.330 (3)
C5—H5A	0.9300	C22—H22A	0.9300
C6—C7	1.388 (3)	C23—C24	1.425 (3)
C7—C8	1.379 (3)	C23—H23A	0.9300
C7—H7A	0.9300	C24—C25	1.388 (3)
C8—C13	1.422 (3)	C24—C33	1.416 (3)
C8—C9	1.432 (3)	C25—C26	1.371 (3)
C9—C10	1.349 (4)	C25—H25A	0.9300
C9—H9A	0.9300	C26—C27	1.372 (3)
C10—C11	1.394 (4)	C26—H26A	0.9300
C10—H10A	0.9300	C27—C28	1.391 (3)
C11—C12	1.347 (3)	C27—H27A	0.9300
C11—H11A	0.9300	C28—C33	1.413 (3)
C12—C13	1.421 (3)	C28—C29	1.417 (3)
C12—H12A	0.9300	C29—C30	1.346 (3)
C13—C14	1.398 (3)	C29—H29A	0.9300
C14—C15	1.501 (3)	C30—C31	1.436 (2)
C15—C16	1.452 (3)	C30—H30A	0.9300
C16—C17	1.328 (3)	C31—C32	1.419 (2)
C16—H16A	0.9300	C32—C33	1.418 (2)
			
C14—C1—C2	122.1 (2)	C18—C17—H17A	115.9
C14—C1—C6	119.2 (2)	C19—C18—C31	118.81 (18)
C2—C1—C6	118.6 (2)	C19—C18—C17	120.35 (17)
C3—C2—C1	120.7 (3)	C31—C18—C17	120.83 (17)
C3—C2—H2A	119.7	C20—C19—C18	121.75 (19)
C1—C2—H2A	119.7	C20—C19—H19A	119.1
C2—C3—C4	120.5 (3)	C18—C19—H19A	119.1
C2—C3—H3A	119.7	C19—C20—C21	121.17 (18)
C4—C3—H3A	119.7	C19—C20—H20A	119.4
C5—C4—C3	120.8 (3)	C21—C20—H20A	119.4
C5—C4—H4A	119.6	C20—C21—C32	118.97 (18)
C3—C4—H4A	119.6	C20—C21—C22	122.38 (19)
C4—C5—C6	121.1 (3)	C32—C21—C22	118.65 (18)
C4—C5—H5A	119.5	C23—C22—C21	121.3 (2)
C6—C5—H5A	119.5	C23—C22—H22A	119.3
C7—C6—C5	122.7 (2)	C21—C22—H22A	119.3
C7—C6—C1	118.9 (2)	C22—C23—C24	122.0 (2)
C5—C6—C1	118.3 (2)	C22—C23—H23A	119.0
C8—C7—C6	122.3 (2)	C24—C23—H23A	119.0
C8—C7—H7A	118.8	C25—C24—C33	119.0 (2)
C6—C7—H7A	118.8	C25—C24—C23	122.8 (2)
C7—C8—C13	119.2 (2)	C33—C24—C23	118.20 (18)
C7—C8—C9	122.6 (2)	C26—C25—C24	121.0 (2)
C13—C8—C9	118.2 (2)	C26—C25—H25A	119.5
C10—C9—C8	120.8 (2)	C24—C25—H25A	119.5
C10—C9—H9A	119.6	C25—C26—C27	120.8 (2)
C8—C9—H9A	119.6	C25—C26—H26A	119.6
C9—C10—C11	120.6 (2)	C27—C26—H26A	119.6
C9—C10—H10A	119.7	C26—C27—C28	120.6 (2)
C11—C10—H10A	119.7	C26—C27—H27A	119.7
C12—C11—C10	121.0 (3)	C28—C27—H27A	119.7
C12—C11—H11A	119.5	C27—C28—C33	119.35 (19)
C10—C11—H11A	119.5	C27—C28—C29	122.59 (19)
C11—C12—C13	120.9 (2)	C33—C28—C29	118.05 (17)
C11—C12—H12A	119.5	C30—C29—C28	121.96 (18)
C13—C12—H12A	119.5	C30—C29—H29A	119.0
C14—C13—C12	122.4 (2)	C28—C29—H29A	119.0
C14—C13—C8	119.1 (2)	C29—C30—C31	121.82 (19)
C12—C13—C8	118.5 (2)	C29—C30—H30A	119.1
C1—C14—C13	121.20 (19)	C31—C30—H30A	119.1
C1—C14—C15	119.54 (18)	C18—C31—C32	119.28 (16)
C13—C14—C15	119.13 (19)	C18—C31—C30	123.45 (17)
O1—C15—C16	121.8 (2)	C32—C31—C30	117.26 (16)
O1—C15—C14	120.8 (2)	C21—C32—C33	119.57 (17)
C16—C15—C14	117.37 (16)	C21—C32—C31	120.00 (17)
C17—C16—C15	122.12 (19)	C33—C32—C31	120.43 (16)
C17—C16—H16A	118.9	C28—C33—C24	119.28 (17)
C15—C16—H16A	118.9	C28—C33—C32	120.46 (17)
C16—C17—C18	128.28 (19)	C24—C33—C32	120.26 (17)
C16—C17—H17A	115.9		
			
C14—C1—C2—C3	178.7 (2)	C17—C18—C19—C20	−179.72 (19)
C6—C1—C2—C3	0.1 (3)	C18—C19—C20—C21	0.4 (3)
C1—C2—C3—C4	−0.5 (4)	C19—C20—C21—C32	−1.2 (3)
C2—C3—C4—C5	0.8 (4)	C19—C20—C21—C22	178.09 (19)
C3—C4—C5—C6	−0.6 (4)	C20—C21—C22—C23	−179.9 (2)
C4—C5—C6—C7	−178.7 (2)	C32—C21—C22—C23	−0.6 (3)
C4—C5—C6—C1	0.1 (4)	C21—C22—C23—C24	−0.2 (3)
C14—C1—C6—C7	0.3 (3)	C22—C23—C24—C25	−179.0 (2)
C2—C1—C6—C7	178.97 (19)	C22—C23—C24—C33	0.6 (3)
C14—C1—C6—C5	−178.49 (19)	C33—C24—C25—C26	−0.6 (3)
C2—C1—C6—C5	0.1 (3)	C23—C24—C25—C26	179.0 (2)
C5—C6—C7—C8	178.2 (2)	C24—C25—C26—C27	0.7 (3)
C1—C6—C7—C8	−0.6 (3)	C25—C26—C27—C28	−0.3 (3)
C6—C7—C8—C13	0.7 (3)	C26—C27—C28—C33	−0.1 (3)
C6—C7—C8—C9	−178.1 (2)	C26—C27—C28—C29	179.52 (19)
C7—C8—C9—C10	179.0 (2)	C27—C28—C29—C30	−179.52 (19)
C13—C8—C9—C10	0.2 (3)	C33—C28—C29—C30	0.1 (3)
C8—C9—C10—C11	0.3 (4)	C28—C29—C30—C31	−1.2 (3)
C9—C10—C11—C12	−0.6 (4)	C19—C18—C31—C32	−1.7 (3)
C10—C11—C12—C13	0.2 (3)	C17—C18—C31—C32	179.07 (16)
C11—C12—C13—C14	−178.52 (19)	C19—C18—C31—C30	179.00 (17)
C11—C12—C13—C8	0.3 (3)	C17—C18—C31—C30	−0.2 (3)
C7—C8—C13—C14	−0.5 (3)	C29—C30—C31—C18	−179.86 (18)
C9—C8—C13—C14	178.36 (18)	C29—C30—C31—C32	0.9 (3)
C7—C8—C13—C12	−179.37 (19)	C20—C21—C32—C33	−179.70 (17)
C9—C8—C13—C12	−0.5 (3)	C22—C21—C32—C33	1.0 (3)
C2—C1—C14—C13	−178.78 (18)	C20—C21—C32—C31	0.5 (3)
C6—C1—C14—C13	−0.2 (3)	C22—C21—C32—C31	−178.81 (17)
C2—C1—C14—C15	−2.9 (3)	C18—C31—C32—C21	0.9 (2)
C6—C1—C14—C15	175.70 (17)	C30—C31—C32—C21	−179.74 (16)
C12—C13—C14—C1	179.10 (18)	C18—C31—C32—C33	−178.83 (16)
C8—C13—C14—C1	0.3 (3)	C30—C31—C32—C33	0.5 (2)
C12—C13—C14—C15	3.2 (3)	C27—C28—C33—C24	0.2 (3)
C8—C13—C14—C15	−175.62 (17)	C29—C28—C33—C24	−179.45 (16)
C1—C14—C15—O1	91.0 (2)	C27—C28—C33—C32	−179.13 (17)
C13—C14—C15—O1	−93.0 (2)	C29—C28—C33—C32	1.2 (2)
C1—C14—C15—C16	−87.9 (2)	C25—C24—C33—C28	0.1 (3)
C13—C14—C15—C16	88.1 (2)	C23—C24—C33—C28	−179.46 (17)
O1—C15—C16—C17	−172.6 (2)	C25—C24—C33—C32	179.47 (17)
C14—C15—C16—C17	6.3 (3)	C23—C24—C33—C32	−0.1 (3)
C15—C16—C17—C18	176.81 (19)	C21—C32—C33—C28	178.70 (16)
C16—C17—C18—C19	17.2 (3)	C31—C32—C33—C28	−1.5 (2)
C16—C17—C18—C31	−163.6 (2)	C21—C32—C33—C24	−0.6 (2)
C31—C18—C19—C20	1.1 (3)	C31—C32—C33—C24	179.16 (16)

### 1-(Anthracen-9-yl)-3-(pyren-1-yl)prop-2-en-1-one (II) Hydrogen-bond geometry (Å, º)

Cg3, Cg4 and Cg5 are the centroids of the C18–C13, C21–C24/C32/C33 and C18–C21/C31/C32 rings

**Table:**

*D*—H···*A*	*D*—H	H···*A*	*D*···*A*	*D*—H···*A*
C25—H25*A*···*Cg*3^i^	0.93	2.82	3.7220 (3)	164
C26—H26*A*···*Cg*5^ii^	0.93	2.81	3.6050 (3)	144
C27—H27*A*···*Cg*4^ii^	0.93	2.95	3.6520 (3)	134

Symmetry codes: (i) −*x*+1, −*y*+1, −*z*+1; (ii) *x*, −*y*+1/2, *z*−3/2.
